# Health-related quality of life in patients with rheumatoid arthritis

**DOI:** 10.1186/s41927-019-0080-9

**Published:** 2019-08-14

**Authors:** Wanruchada Katchamart, Pongthorn Narongroeknawin, Wanwisa Chanapai, Phakhamon Thaweeratthakul

**Affiliations:** 1 Division of Rheumatology, Department of Medicine, Faculty of Medicine Siriraj Hospital, Mahidol University, 8th floor Asadang building, 2 Wanglang road, Bangkok-noi, Bangkok, 10700 Thailand; 2 Division of Rheumatology, Department of Medicine, Phramongkutklao Hospital and College of Medicine, Bangkok, Thailand; 3 Division of Clinical Trials, Research Department, Faculty of Medicine Siriraj Hospital, Bangkok, Thailand

**Keywords:** Rheumatoid arthritis, Health-related quality of life, EQ-5D, DAS28, HAQ, Depression, Anxiety

## Abstract

**Background:**

Rheumatoid arthritis (RA) is a chronic systemic autoimmune disease that primarily affects joints with some extraarticular involvement. If inappropriately treated, it usually results in persistent joint pain, irreversible deformities, and functional disability, leading to poor quality of life. Our objective was to evaluate health-related quality of life (HRQoL) and related factors in patients with RA.

**Methods:**

Four hundred sixty-four patients from the Rheumatoid Arthritis registries of Siriraj and Phramongkutklao teaching hospitals were enrolled. Sociodemographic, clinical and laboratory data related to disease activity, and functional status were collected. HRQoL was assessed using the Thai version of EuroQol five dimensional questionnaire (EQ-5D) and EQ global health visual analogue scale (EQ VAS). Univariate and multivariate analyses were employed to identify factors related to HRQoL.

**Results:**

Eighty-five percent were female with a mean age ± SD of 59.15 ± 11.43 years and a mean disease duration ± SD of 11.53 ± 8.3 years. The mean educational level ± SD was 9.42 ± 5.21 years. Almost half were unemployed or retired (47%). They had moderate disease activity (mean cumulative DAS28 ± SD, 3.5 ± 0.8) and mild functional impairment (mean HAQ ± SD, 0.70 ± 0.68). The mean EQ-5D ± SD (0–1) was 0.87 ± 0.13 and mean EQ VAS ± SD (0–10) was 7.94 ± 1.7. Based on the EQ-5D domain, 49% reported that they had no problem with mobility, 83% had no difficulties with self-care, 65% had no difficulties with usual activity, 30% had no pain or discomfort, and 61% had no depression or anxiety. The relationship between problems of each dimension in EQ-5D significantly increased according to severity of RA assessed by the Disease Activity Score (DAS) 28 and Health Assessment Questionnaire (HAQ) (*p* <  0.01). In multivariate analyses, high cumulative disease activity, functional disability, depression, and anxiety were negatively associated with EQ-5D *(adjusted R*^*2*^ *0.38, p <  0.001)* and EQ VAS *(adjusted R* ^*2*^ *0.19, p <  0.001).*

**Conclusion:**

Disease severity and psychological disturbance have a negative impact on quality of life in patients with RA. These factors should be considered in management of RA patients to improve the standard of care.

**Electronic supplementary material:**

The online version of this article (10.1186/s41927-019-0080-9) contains supplementary material, which is available to authorized users.

## Background

Rheumatoid arthritis (RA) is a chronic systemic autoimmune disease that primarily affects joints with some extraarticular involvement. If inappropriately treated, it usually results in persistent joint pain, irreversible deformities, and functional disability, leading to poor quality of life. RA can be a substantial burden to those afflicted and their families. Therefore, the aim of treatment is to suppress inflammation, prevent joint damage, and resume normal joint function. An early, aggressive, treat-to-target aimed at remission approach is recommended to achieve this goal [1–3]. The treatment outcome assessment is usually focused on reduced disease activity and improved functional status, such as, number of tender and swollen joints, pain score, global disease activity score assessed by physician or patients, and inflammatory markers. However, health-related quality of life (HRQoL) is an important outcome that should be considered in the management of chronic illnesses including RA. HRQoL measures reflect a subjective evaluation of the following key dimensions: the physical dimension (pain and deterioration of physical functioning), the psychological dimension (anxiety and depression), the intellectual or cognitive dimension (attention and memory), and the social dimension (self-esteem and interpersonal relationships) [4]. It is an individual’s perceived physical and mental health over time. Many HRQoL instruments, both generic and specific, are used in clinical trials. The EuroQoL five dimensional questionnaire (EQ-5D) is one of the most widely used HRQoL instruments in health related research [5, 6]. It is a standardized instrument developed by the EuroQol Group as a measure of HRQoL that can be used in a wide range of health conditions and treatments. It has been shown in previous studies that quality of life in patients with RA varies among different population [7–9]. Currently, there is no data related to HRQoL in Thai population with RA. Thus, the purpose of this study was to evaluate the HRQoL and its relationship to patient characteristics in Thai patients with rheumatoid arthritis.

## Methods

### Study population

All eligible patients from the Rheumatoid Arthritis registries of Siriraj and Phramongkutklao hospitals were consecutively enrolled from September 2016 to March 2018. The Siriraj Rheumatoid Arthritis (SiRA) registry is a prospective cohort established in 2011 by the outpatient service of the Division of Rheumatology, Department of Medicine, Faculty of Medicine, Siriraj Hospital, Bangkok, Thailand [10]. The Thai Army Rheumatoid Arthritis Cohort (TARAC) is an observational, real-world cohort of patients with RA recruited at the Rheumatology Clinic of Phramongkutklao Hospital since 1990 [11]. Patients were eligible if they were 18 years old or older, had been diagnosed with RA according to the American College of Rheumatology 1987 revised criteria for the classification of RA [12] or the 2010 Rheumatoid arthritis classification criteria [13], had at least one follow-up visit, and had complete relevant data. They were excluded if they were diagnosed with overlapping syndrome with other rheumatic or autoimmune diseases. Signed informed consent was obtained from all participants prior to enrollment.

### Study variables

Baseline characteristics, co-morbid conditions, disease activity, and functional status were recorded. Disease activity was measured at baseline and every 3–4 month of follow-up visit using the Disease activity score28 (DAS28) [14] Functional status at current visit was measured using the Thai version of Health assessment questionnaire (HAQ) [15]. Cumulative disease activity were calculated using the time-adjusted mean DAS28, which is the area under a curve (AUC) of DAS28 plotted against time, divided by the total length of time from first to last measurement. Depression and anxiety at current visit were assessed by the Thai version of the Hospital Anxiety and Depression Scale (Thai HADS) [16]. A score of 8 or higher for either type of mood disorder indicated the presence of some degree of anxiety or depression. The Thai version of the EuroQoL five dimensional questionnaire (EQ-5D) at current visit was used for the assessment of HRQoL [17]. The EQ-5D consists of a descriptive system and the EQ visual analogue scale (VAS). The descriptive system comprises five dimensions: mobility, self-care, usual activities, pain/discomfort and anxiety/depression. The EQ VAS records the patient’s self-rated health on a 0–10 vertical VAS.

### Statistical analyses

Descriptive statistical analysis was used for patient characteristics and clinical variables. Demographic and baseline characteristics were described using mean and standard deviation (SD) or median and range for continuous data and number and percentage for categorical data. Comparisons of demographic and characteristics between groups were performed using the Chi-square test or Fisher’s exact test as appropriate for categorical data and student-t test or Kruskal–Wallis tests as appropriate for continuous data. Univariate and multiple linear regression analyses were performed to identify independent factors associated with EQ-5D and EQ VAS. Factors that were identified to be different between groups with a *p*-value of less than 0.2 in univariate analysis were included in a multivariate analysis. A p-value of less than 0.05 was considered statistically significant. All analyses were performed using the Statistical Package for the Social Science (SPSS) 20.0.

This study was conducted in accordance with the ethical principles of the Declaration of Helsinki and adhered to the principles outlined in the Guideline for Good Clinical Practice International Conference on Harmonization (ICH) Tripartite Guideline (January 1997). The study protocol was approved by the local ethics committees.

## Results

Among the 464 RA patients, 85% were female with a mean age ± SD of 59.15 ± 11.43 years and a mean disease duration ± SD of 11.53 ± 8.3 years. The mean duration of follow-up ± SD was 4.28 ± 2.29 years. The mean educational level ± SD was 9.42 ± 5.21 years. Almost half were unemployed or retired (47%). They had moderate disease activity (mean cumulative DAS28 ± SD, 3.5 ± 0.8) and mild functional impairment (mean HAQ ± SD, 0.70 ± 0.68). In terms of serology, 75% had rheumatoid factor and 72% had anti-citrullinated protein antibody (ACPA). Most (84%) had radiographic hand or feet erosion at baseline. Comorbid conditions were as follows: hypertension (41%), followed by dyslipidemia (39%), diabetes mellitus (10%), coronary artery disease (2.6%), and cerebrovascular disease (1.7%). Depression and anxiety were recognized in 8.4 and 9.3%, respectively *(*Table 1*).*

**Table 1:** Baseline characteristics of 464 patients with RA

Characteristics	N/total (%) or Mean ± SD
Age, years	59.15 ± 11.43
Women	395/464 (85.1)
Smoking	46/464 (9.9)
Alcohol drinking	105/464 (22.6)
Education, years	9.42 ± 5.21
Unemployed or retired	221/464 (47.6)
Disease duration, years	11.53 ± 8.3
Follow-up duration, years	4.28 ± 2.29
DAS28 (0–9)	3.50 ± 0.81
HAQ (0–3)	0.70 ± 0.68
Rheumatoid factor	334/447 (74.7)
ACPA	276/382 (72.3)
Hand or feet erosion	357/427 (83.6)
Diabetes mellitus	46/464 (9.9)
Hypertension	188/464 (40.5)
Dyslipidemia	182/464 (39.2)
Cerebrovascular disease	8/464 (1.7)
Coronary artery disease	12/464 (2.6)
Depression	39/464 (8.4)
Anxiety	43/464 (9.3)

Abbreviation: - *DAS28* Disease activity score 28, *HAQ* Health assessment questionnaire, *ACPA* anti-citrullinated peptide antibodies

### Health-related quality of life

The mean EQ-5D ± SD was 0.87 ± 0.13 and the mean EQ VAS ± SD was 7.94 ± 1.7. Based on EQ-5D domain (Table 2), 83% participants reported that they had no problem with self-care, whereas 65% had no problem with usual activity, and 61% had no depression or anxiety. Almost half (49%) had no problem with mobility. Only 30% had no pain or discomfort.

**Table 2:** Descriptive statistics of EQ-5D domains

Domain	No problem	Slight	Moderate	Severe	Unable
Mobility, N (%)	225 (48.5)	152 (32.8)	64 (13.8)	20 (4.3)	3 (0.6)
Self-care, N (%)	386 (83.2)	54 (11.6)	19 (4.1)	3 (0.6)	2 (0.4)
Usual activities N (%)	300 (64.7)	117 (25.2)	38 (8.2)	8 (1.7)	1 (0.2)
Pain/Discomfort N (%)	137 (29.5)	238 (51.3)	82 (17.7)	5 (1.1)	2 (0.4)
Anxiety/Depression N (%)	284 (61.2)	149 (32.1)	25 (5.4)	5 (1.1)	1 (0.2)

Regarding relationship between patient characteristics and domains of EQ-5D (Additional file 1: Table supplement), the proportion of any problem with mobility were significantly higher in patients with long-standing disease (≥ 10 years), unemployment, presence of ACPA, coronary artery disease, high cumulative disease activity (cumulative DAS28 > 2.6), functional impairment (HAQ >  0.5), and depression.

**Fig. 1:**
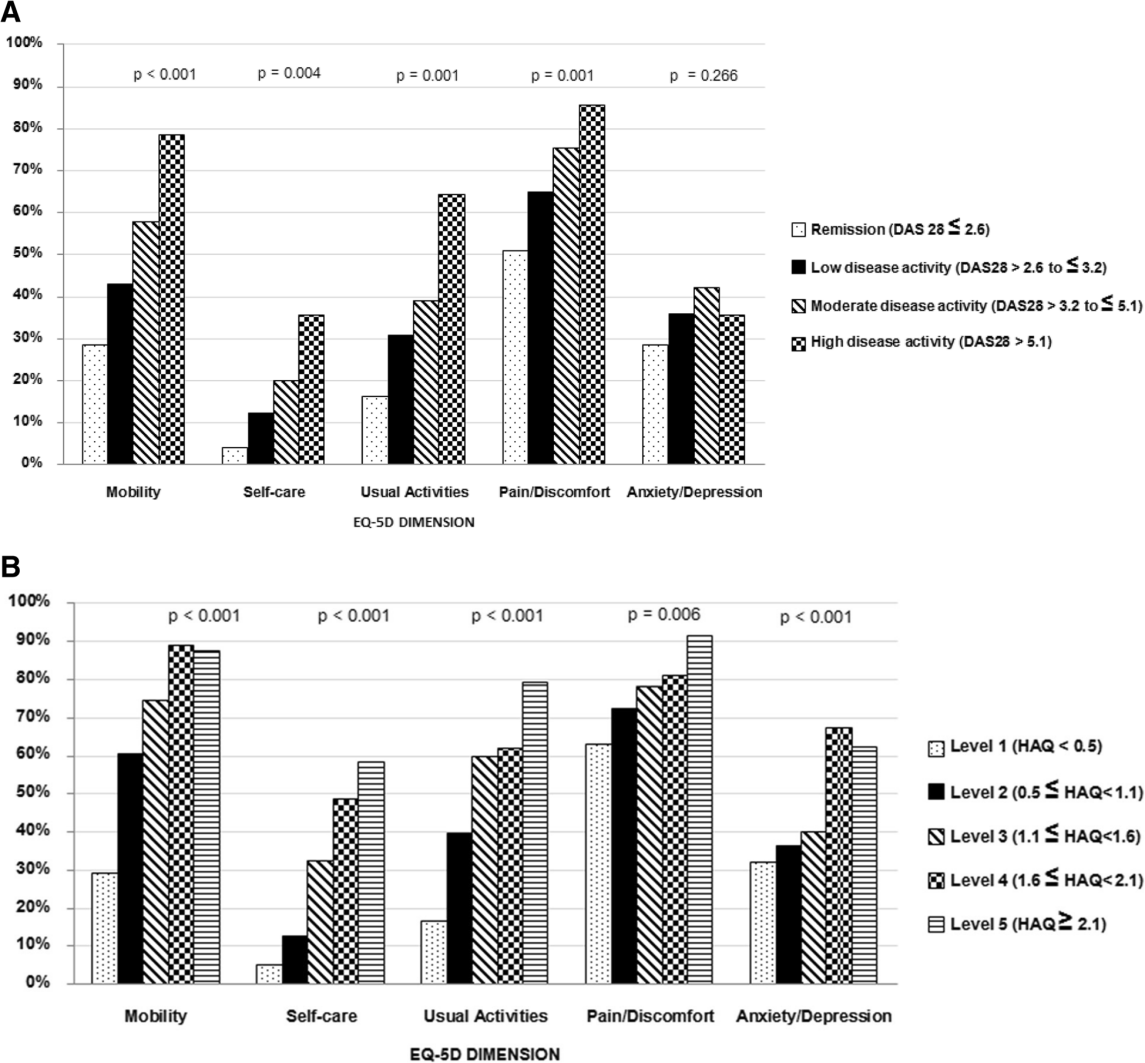
Patients with problem in each dimension of EQ-5D according to disease severity measured by the disease activity score 28 (DAS28) (a) and the health assessment questionnaire (HAQ) (b)

Patients with impaired functional status (HAQ >  0.5) had more significant problem with all domains of EQ-5D. Similar to functional status, more patients with active disease (DAS28 > 2.6) also had significantly problems with all domains of EQ-5D, except for depression and anxiety. We explored the impact of disease activity and functional disability on HRQoL and found that the higher disease activity, the lower EQ-5D and EQ VAS (*p* <  0.001). These similar relationships were also found between functional status and EQ-5D/EQ VAS and EQ VAS scores (*p* <  0.001) *(*Table 3*).*. Furthermore, the relationship between problems of each dimension in EQ-5D significantly increased according to severity of RA assessed by DAS28 and HAQ (*p* <  0.01), except for depression/anxiety (Fig. 1).

**Table 3:** Relationship between disease activity and functional status and EQ-5D

	Mean EQ-5D ± SD	Mean EQ VAS ± SD
Disease activity		
Remission	0.93 ± 0.10	8.49 ± 1.3
Low (DAS28 > 2.6 to ≤3.2)	0.89 ± 0.12	8.17 ± 1.51
Moderate (DAS28 > 3.2 to ≤5.1)	0.85 ± 0.14	7.77 ± 1.8
High (DAS28 > 5.1)	0.79 ± 0.19	7.23 ± 1.85
Functional status		
HAQ < 0.5	0.92 ± 0.09	8.4 5 ± 1.45
0.5 ≤ HAQ < 1.1	0.88 ± 0.09	7.97 ± 1.62
1.1 ≤ HAQ < 1.6	0.80 ± 0.16	7.4 ± 1.71
1.6 ≤ HAQ < 2.1	0.72 ± 0.19	6.81 ± 1.78
HAQ ≥ 2.1	0.72 ± 0.16	6.17 ± 1.79

Abbreviation: - *DAS28* Disease activity score 28, *HAQ* Health assessment questionnaire

### Factor associated with EQ-5D and EQ VAS

Patients with unemployment, high cumulative disease activity, functional impairment, coronary artery syndrome, depression, and anxiety had significantly lower EQ-5D scores in univariate analysis (*p* <  0.05) (Table 4). All these factors, except unemployment, were independently associated with EQ-5D in multivariate analysis (Table 5). The adjusted *R*^2^ was 0.376 (F = 35.905, *p* <  0.001).

**Table 4:** Association between EQ-5D score, global health (VAS), and patient characteristics

	EQ-5D score				Global health (VAS)			
	Mean ± SD	*P* value	beta	SE	Mean ± SD	*P* value	beta	SE
Age								
● > 60 years	0.86 ± 0.13	0.184	−0.016	0.012	7.8 ± 1.76	0.039	−3.260	1.571
● ≤ 60 years	0.88 ± 0.13				8.1 ± 1.62			
Sex								
● Woman	0.87 ± 0.14	0.244	−0.020	0.017	7.92 ± 1.7	0.681	−0.912	2.218
● Man	0.89 ± 0.11				8.01 ± 1.71			
Smoking								
● Yes	0.88 ± 0.11	0.566	0.012	0.021	8.08 ± 1.55	0.559	1.546	2.640
● No	0.87 ± 0.13				7.92 ± 1.71			
Alcohol drinking								
● Yes	0.87 ± 0.13	0.842	0.003	0.015	7.91 ± 1.6	0.826	−0.415	1.886
● No	0.87 ± 0.13				7.95 ± 1.73			
Education								
● < 6 years	0.86 ± 0.14	0.451	−0.010	0.013	7.71 ± 1.72	0.022	−3.711	1.619
● ≥ 6 years	0.87 ± 0.13				8.08 ± 1.67			
Disease duration								
● ≥ 10 years	0.86 ± 0.14	0.529	−0.008	0.012	7.83 ± 1.63	0.195	−2.044	1.576
● < 10 years	0.87 ± 0.12				8.04 ± 1.76			
Unemployed or retired								
● Yes	0.85 ± 0.14	0.019	−0.029	0.012	7.85 ± 1.71	0.316	−1.585	1.578
● No	0.88 ± 0.12				8.01 ± 1.68			
DAS 28								
● Non-remission (DAS28 > 2.6)	0.86 ± 0.13	< 0.001	−0.064	0.020	7.87 ± 1.73	0.017	−6.137	2.552
● Remission (DAS28 ≤ 2.6)	0.93 ± 0.10				8.49 ± 1.3			
HAQ								
● > 0.5	0.81 ± 0.15	< 0.001	−0.106	0.011	7.42 ± 1.8	< 0.001	−9.693	1.516
● ≤ 0.5	0.92 ± 0.09				8.39 ± 1.46			
Rheumatoid factor								
● Yes	0.87 ± 0.13	0.823	−0.003	0.014	7.9 ± 1.75	0.695	−0.732	1.866
● No	0.87 ± 0.14				7.97 ± 1.61			
ACPA								
● Yes	0.87 ± 0.13	0.323	−0.015	0.015	7.98 ± 1.68	0.645	0.887	1.926
● No	0.88 ± 0.13				7.89 ± 1.71			
Hand or feet erosion								
● Yes	0.86 ± 0.14	0.315	−0.018	0.017	7.88 ± 1.7	0.554	−1.308	2.208
● No	0.88 ± 0.11				8.01 ± 1.64			
Diabetes mellitus								
● Yes	0.89 ± 0.09	0.101	0.025	0.021	8.0 ± 2.04	0.784	0.725	2.641
● No					7.93 ± 1.66			
Hypertension								
● Yes	0.86 ± 0.14	0.294	−0.013	0.013	8.0. ± 1.74	0.498	1.088	1.607
● No	0.87 ± 0.13				7.89 ± 1.67			
Dyslipidemia								
● Yes	0.87 ± 0.13	0.801	0.003	0.013	8.1 ± 1.71	0.105	2.621	1.612
● No	0.87 ± 0.13				7.83 ± 1.69			
Stroke								
● Yes	0.89 ± 0.08	0.712	0.017	0.047	7.63 ± 2.49	0.601	−3.173	6.061
● No	0.87 ± 0.13				7.9.4 ± 1.68			
Coronary artery disease								
● Yes	0.79 ± 0.17	0.048	−0.077	0.039	7.46 ± 2.5	0.323	−4.912	4.967
● No	0.87 ± 0.13				7.95 ± 1.69			
Depression								
● Yes	0.74 ± 0.21	< 0.001	−0.138	0.021	6.78 ± 1.9	< 0.001	−12.608	2.783
● No	0.88 ± 0.12				8.04 ± 1.64			
Anxiety								
● Yes	0.73 ± 0.20	< 0.001	−0.154	0.020	6.83 ± 1.95	< 0.001	−12.248	2.661
● No	0.88 ± 0.11				8.05 ± 1.63			

Abbreviation:- *EQ-5D* EuroQol five dimensional questionnaire, *VAS* Visual analogue scale, *DAS28* Disease activity score28, *HAQ* Health assessment questionnaire, *ACPA* anti-citrullinated peptide antibodies, *SD* standard deviation, *SE* standard error

**Table 5:** Multivariate analysis of relationship between EQ-5D, EQ global health (VAS), and patient characteristics

Variables	EQ-5D score			EQ Global health (VAS)		
	Beta	95% CI of beta		Beta	95% CI of beta	
		lower	upper		lower	upper
Age	0.001	0.0001	0.002	−0.051	− 0.192	0.091
Unemployed or retired	−0.014	− 0.306	0.007			
Education > 6 years				−1.105	−4.329	2.120
Disease duration ≥10 years				0.472	−2.392	3.335
DAS28	−0.014*	−0.027	−0.001	−1.889*	−3.730	− 0.048
HAQ	−0.090*	− 0.106	−0.075	−8.088*	−10.403	−5.773
DM	0.017	−0.016	0.049			
CAD	−0.033*	0.294	−0.094			
Dyslipidemia				2.109	−0.929	5.146
Depression	−0.077*	−0.115	− 0.039	−7.563*	−13.117	−2.008
Anxiety	−0.087*	− 0.124	−0.050	−5.921*	− 11.293	− 0.548
Constant	1.124	1.043	1.205	108.626	96.971	120.58

Abbreviation:- *EQ-5D* EuroQol five dimensional questionnaire, *VAS* Visual analogue scale, *DAS28* Disease activity score28, *HAQ* Health assessment questionnaire, *DM* Diabetes mellitus, *CAD* Coronary artery disease, *SE* standard error

*p <  0.05

Patients with age of more than 60 years, low education (< 6 years), high cumulative disease activity, functional impairment, depression, and anxiety had significantly lower global health scores in univariate analysis (*p* <  0.05) (Table 4). However, only high cumulative disease activity, functional impairment, depression, and anxiety were independently associated with EQ VAS global health scores in multivariate analysis (Table 5). The adjusted R^2^ was 0.189 (F = 14.447, *p* <  0.001).

## Discussion

This study demonstrated that the RA disease activity, functional status, depression, and anxiety have a significant impact on HRQoL in the Thai population. The mean EQ-5D ± SD was 0.87 ± 0.13 meaning that Thai patients with RA perceived their current health at approximately 87% of perfect health. These findings were from a cross-sectional assessment of HRQoL. HRQoL can vary over time. However, this score was higher than that of Japanese population (mean EQ-5D 0.76 ± 0.18) [8], Korean population (mean EQ-5D 0.70 ± 0.26) [9], Danish population (mean EQ-5D ± SD 0.73 ± 0.19) [18], and Spanish population (mean EQ-5D ± SD 0.63 ± 0.20) [7] despite having a comparable age group (55–60 years old) and disease duration (10–11 years). Spanish patients had more functional impairment (mean HAQ ± SD 1.02 ± 0.78), so the quality of life was much lower than that of Thai. Thai patients did report higher disease activity with mean DAS28 ± SD of 3.5 ± 0.8, representing moderate disease activity, whereas Korean and Japanese patients had low disease activity with mean DAS28 ± SD of 2.85 ± 1.13 and median DAS28 (range) 3.2 (2.4–4.0), respectively. Furthermore, Thai patients had more functional disability (mean HAQ ± SD 0.7 ± 0.68), as compared to Korean (mean HAQ ± SD 0.62 ± 0.66), Japanese [median HAQ (range) 0.5 (0–1.25)], and Danish [median HAQ (range) 0.63 (0.25–1.25)]. These findings may reflect differences in patient s’ perception, beliefs, culture, socioeconomic status, health care system, as well as, social structure.

Similar to previous studies [7–9, 18], this study also demonstrated that the HRQoL of the RA patients is significantly associated with disease activity and functional status. Furthermore, we showed that depression and anxiety are additional factors related to quality of life. All these factors found in our studies are relevant and reasonable because three out of 5 domains in EQ-5D measure mobility, self-care, and usual activities that are also included in the Thai HAQ, and depression and anxiety are also an item in EQ-5D, as well. Therefore it would be valuable to included EQ-5D as one of the patient-reported outcome measures routinely used in clinical trial and daily practice due to its comprehensiveness, relevance, and feasibility.

Compared to the Thai patients with ankylosing spondylitis (AS), AS patients had a lower EQ-5D and EG-VAS with mean ± SD of 0.75 ± 0.2 and 6.9 ± 1.9, respectively, despite having mild disease activity (mean BASDAI ± SD 3.2 ± 3.7) and mild functional impairment (mean HAQ ± SD 0.4 ± 0.9) [19]. Most of AS patients were male (61%) in working age (mean age ± SD 40 ± 12 years). They may have to perform activity in their work more than basic activity of daily living. Workplace activities are not captured by HAQ. Thus they may have a higher expectation than that of patients with RA, who were mostly female, elderly, and unemployed.

When compared to other chronic illnesses in the Thai population, patients with RA had a higher HRQoL than end-stage renal disease (ESRD) patients undergoing peritoneal dialysis (mean EQ-5D ± SD 0.65 ± 0.23) [20] and those with ischemic heart disease (mean EQ-5D ± SD 0.75 ± 0.20) [21]. Although most ESRD patients had fewer problems with mobility (no problem 81% vs. 49%), they had a significant problem with performing usual activity (63% vs.36%) and high proportion of depression and anxiety (52% vs. 40%) than patients with RA [20]. Patients with RA had comparable HRQoL, when compared to patients with chronic epilepsy, who were seizure-free (mean EQ-5D ± SD 0.82 ± 0.15, mean EQ-VAS 7.89 ± 1.45) with different dimensions affecting HRQoL. Even though patients with chronic epilepsy had fewer problems with physical activities and pain, they had more psychological problem than patients with RA (moderate to severe problem 42% vs. 7%) [22].

We used longitudinal data to demonstrate the impact of cumulative disease activity on HRQoL, supporting the concept that a tight control strategy aiming at ‘sustained’ remission or at least low disease activity can improve function leading to better quality of life [3]. However, we cross-sectionally assessed EQ-5D once for current analyses, so we cannot demonstrate the longitudinal relationship between improvement of HRQoL and disease activity eventually.

## Conclusions

Thai patients with RA reported a higher HRQoL than other RA populations. High cumulative disease activity, functional impairment, depression, and anxiety are negatively associated with HRQoL. To improve the quality of life of RA patients in daily practice, all of these factors should be taken into account in a holistic approach. Further studies should explore the relationship between improvement of these factors and HRQoL.

## Additional file

Additional file 1: Table Supplement Relationship between the five domains of the EQ-5D and patient characteristics. (DOCX 54 kb)

## Data Availability

The datasets used and/or analysed during the current study are available from the corresponding author on reasonable request.

## Abbreviations

ACPA: Anti-citrullinated protein antibody
AS: Ankylosing spondylitis
AUC: Area under a curve
BASDAI: Bath assessment of disease activity index
CAD: Coronary artery disease
DAS28: Disease activity score 28
DM: Diabetes mellitus
EQ VAS: EQ global health visual analogue scale
EQ-5D: EuroQol five dimensional questionnaire
ESRD: End stage renal disease
HADS: Hospital Anxiety and Depression Scale
HAQ: Health Assessment Questionnaire
HRQoL: Health-related quality of life
RA: Rheumatoid arthritis
SD: Standard deviation
SE: Standard error
