# Supplementary material for: Health-related quality of life in patients with rheumatoid arthritis
Source: BMC Rheumatol. 2019 Aug 14;3:34. doi: 10.1186/s41927-019-0080-9 (PMC6694487; doi:10.1186/s41927-019-0080-9)
Supplement: Supplementary file 1 — Table Supplement Relationship between the five domains of the EQ-5D and patient characteristics. (DOCX 54 kb) [file 41927_2019_80_MOESM1_ESM.docx]

Table Supplement Relationship between the five domains of the EQ-5D and patient characteristics

| Characteristics | Total | Mobility | | Self-care | | Usual activity | | Pain/discomfort | | Anxiety/ Depression | |
| --- | --- | --- | --- | --- | --- | --- | --- | --- | --- | --- | --- |
|  | n(%) | % any problem | P value | % any problem | P value | % any problem | P value | % any problem | P value | % any problem | P value |
| Age, years | | | | | | | | | | | |
| - ≤ 60 years | 236(50.9) | 114(48.3) | 0.174 | 36(15.3) | 0.362 | 86(36.4) | 0.615 | 170(72.0) | 0.454 | 94(39.8) | 0.641 |
| - >  60 years | 228(49.1) | 125(54.8) |  | 42(18.4) |  | 78(34.2) |  | 157(68.9) |  | 86(37.7) |  |
| Sex | | | | | | | | | | | |
| - Woman | 395(85.1) | 205(51.9) | 0.687 | 70(17.7) | 0.209 | 147(37.2) | 0.044 | 277(70.1) | 0.695 | 150(38.0) | 0.387 |
| - Man | 69(14.9) | 34(49.3) |  | 8(11.6) |  | 17(24.6) |  | 50(72.5) |  | 30(43.5) |  |
| Smoking | | | | | | | | | | | |
| - Yes | 46(9.9) | 24(52.2) | 0.937 | 4(8.7) | 0.121 | 14(30.4) | 0.463 | 33(71.7) | 0.843 | 21(45.7) | 0.314 |
| - No | 418(90.1) | 215(51.4) |  | 74(17.7) |  | 150(35.9) |  | 294(70.3) |  | 159(38.0) |  |
| Alcohol drinking | | | | | | | | | | | |
| - Yes | 105(22.6) | 54(51.4) | 0.985 | 11(10.5) | 0.048 | 37(35.2) | 0.979 | 79(75.2) | 0.224 | 43(41.0) | 0.606 |
| - No | 359(77.4) | 185(51.5) |  | 67(18.7) |  | 127(35.4) |  | 248(69.1) |  | 137(38.2) |  |
| Education | | | | | | | | | | | |
| - <  6 years | 175(37.7) | 93(53.1) | 0.584 | 35(20.0) | 0.153 | 55(31.4) | 0.170 | 109(62.3) | 0.003 | 69(39.4) | 0.827 |
| - ≥ 6 years | 289(62.3) | 146(50.5) |  | 43(14.9) |  | 109(37.7) |  | 218(75.4) |  | 111(38.4) |  |
| Disease duration | | | | | | | | | | | |
| - <  10 years | 233(50.2) | 108(46.4) | 0.026 | 34(14.6) | 0.199 | 91(39.1) | 0.093 | 172(73.8) | 0.113 | 98(42.1) | 0.147 |
| - ≥ 10 years | 231(49.8) | 131(56.7) |  | 44(19.0) |  | 73(31.6) |  | 155(67.1) |  | 82(35.5) |  |
| Unemployed or retired | | | | | | | | | | | |
| - Yes | 221(47.6) | 130(58.8) | 0.003 | 44(19.9) | 0.089 | 82(37.1) | 0.450 | 161(72.9) | 0.285 | 91(41.2) | 0.315 |
| - No | 243(52.4) | 109(44.9) |  | 34(14.0) |  | 82(33.7) |  | 166(68.3) |  | 89(36.6) |  |
| DAS 28 | | | | | | | | | | | |
| - Remission (DAS28 ≤ 2.6) | 49(10.6) | 14(28.6) | 0.001 | 2(4.1) | 0.012 | 8(16.3) | 0.003 | 25(51.0) | 0.002 | 14(28.6) | 0.121 |
| - Non-remission (DAS28 > 2.6) | 415(89.4) | 225(54.2) |  | 76(18.3) |  | 156(37.6) |  | 302(72.8) |  | 166(40.0) |  |
| DAS 28 | | | | | | | | | | | |
| - Low disease activity (DAS28 ≤ 3.2) | 172(37.1) | 67(39.0) | <  0.001 | 17(9.9) | 0.002 | 46(26.7) | 0.003 | 105(61.0) | 0.001 | 58(33.7) | 0.085 |
| - Non-LDA (DAS28 > 3.2) | 292(62.9) | 172(58.9) |  | 61(20.9) |  | 118(40.4) |  | 222(76.0) |  | 122(41.8) |  |
| HAQ | | | | | | | | | | | |
| - ≤ 0.5 | 246(53.0) | 82(33.3) | <  0.001 | 15(6.1) | <  0.001 | 46(18.7) | <  0.001 | 159(64.6) | 0.003 | 83(33.7) | 0.018 |
| - >  0.5 | 218(47.0) | 157(72.0) |  | 63(28.9) |  | 118(54.1) |  | 168(77.1) |  | 97(44.5) |  |
| Rheumatoid factor | | | | | | | | | | | |
| - Positive | 334(74.7) | 178(53.3) | 0.134 | 58(17.4) | 0.725 | 125(37.4) | 0.287 | 236(70.7) | 0.743 | 122(36.5) | 0.260 |
| - Negative | 113(25.3) | 51(45.1) |  | 18(15.9) |  | 36(31.9) |  | 78(69.0) |  | 48(42.5) |  |
| ACPA | | | | | | | | | | | |
| - Positive | 276(72.3) | 147(53.3) | 0.026 | 47(17.0) | 0.648 | 99(35.9) | 0.295 | 190(68.8) | 0.863 | 111(40.2) | 0.340 |
| - Negative | 106(27.7) | 43(40.6) |  | 16(15.1) |  | 32(30.2) |  | 72(67.9) |  | 37(34.9) |  |
| Hand or feet erosion | | | | | | | | | | | |
| - Yes | 357(83.6) | 190(53.2) | 0.352 | 61(17.1) | 0.779 | 127(35.6) | 0.837 | 258(72.3) | 0.268 | 147(41.2) | 0.282 |
| - No | 70(16.4) | 33(47.1) |  | 11(15.7) |  | 24(34.3) |  | 46(65.7) |  | 24(34.3) |  |
| DM | | | | | | | | | | | |
| - Yes | 46(9.9) | 20(43.5) | 0.251 | 6(13.0) | 0.472 | 18(39.1) | 0.571 | 33(71.7) | 0.843 | 15(32.6) | 0.364 |
| - No | 418(90.1) | 219(52.4) |  | 72(17.2) |  | 146(34.9) |  | 294(70.3) |  | 165(39.5) |  |
| Hypertension | | | | | | | | | | | |
| - Yes | 188(40.5) | 105(55.9) | 0.122 | 25(13.3) | 0.095 | 63(33.5) | 0.495 | 136(72.3) | 0.467 | 76(40.4) | 0.551 |
| - No | 275(59.5) | 134(48.6) |  | 53(19.2) |  | 101(36.6) |  | 191(69.2) |  | 104(37.7) |  |
| Dyslipidemia | | | | | | | | | | | |
| - Yes | 182(39.2) | 92(50.5) | 0.740 | 22(12.1) | 0.029 | 59(32.4) | 0.289 | 133(73.1) | 0.323 | 70(38.5) | 0.906 |
| - No | 282(60.8) | 147(52.1) |  | 56(19.9) |  | 105(37.2) |  | 194(68.8) |  | 110(39.0) |  |
| Stroke | | | | | | | | | | | |
| - Yes | 8(1.7) | 3(37.5) | 0.492 | 1(12.5) | 1.0 | 3(37.5) | 1.0 | 8(100) | 0.112 | 3(37.5) | 1.0 |
| - No | 456(98.3) | 236(51.8) |  | 77(16.9) |  | 161(35.3) |  | 319(70.0) |  | 177(38.8) |  |
| CAD | | | | | | | | | | | |
| - Yes | 12(2.6) | 10(83.3) | 0.025 | 3(25.0) | 0.434 | 3(25.0) | 0.552 | 7(58.3) | 0.349 | 8(66.7) | 0.068 |
| - No | 452(97.4) | 229(50.7) |  | 75(16.6) |  | 161(35.6) |  | 320(70.8) |  | 172(38.1) |  |
| Depression | | | | | | | | | | | |
| - Yes | 39(8.4) | 29(74.4) | 0.003 | 15(38.5) | <  0.001 | 24(61.5) | <  0.001 | 30(76.9) | 0.356 | 25(64.1) | 0.001 |
| - No | 425(91.6) | 210(49.4) |  | 63(14.8) |  | 140(32.9) |  | 297(69.9) |  | 155(36.5) |  |
| Anxiety | | | | | | | | | | | |
| - Yes | 43(9.3) | 28(11.7) | 0.061 | 17(39.5) | <  0.001 | 25(58.1) | 0.001 | 36(83.7) | 0.046 | 35(81.4) | < 0.001 |
| - No | 421(90.7) | 211(50.1) |  | 61(14.5) |  | 139(33.0) |  | 291(69.1) |  | 145(34.4) |  |

Abbreviation:- EQ-5D; EuroQol five dimensional questionnaire, VAS; Visual analogue scale, DAS28; Disease activity score28, HAQ; Health assessment questionnaire,ACPA; anti-citrullinated peptide antibodies, DM; Diabetes mellitus, CAD; Coronary artery disease, SD; standard deviation, SE; standard er
